# Postpartum Mood Disorders: Insights into Diagnosis, Prevention, and Treatment

**DOI:** 10.7759/cureus.42107

**Published:** 2023-07-19

**Authors:** Jyotsna Garapati, Shubhada Jajoo, Deeksha Aradhya, Lucky Srivani Reddy, Swati M Dahiphale, Dharmesh J Patel

**Affiliations:** 1 Obstetrics and Gynecology, Jawaharlal Nehru Medical College, Datta Meghe Institute of Higher Education and Research, Wardha, IND

**Keywords:** stigma, healthcare professionals, screening, risk factors, treatment, prevention, diagnosis, postpartum mood disorders

## Abstract

Postpartum mood disorders pose significant challenges to women's mental health and well-being during the postpartum period. This review article provides insights into these disorders' diagnosis, prevention, and treatment. The article begins by discussing the background information on postpartum mood disorders, their significance, and the purpose of understanding them. It then delves into the classification and types of postpartum mood disorders, emphasizing the need for accurate diagnosis and differentiation. Prevalence and incidence rates are explored to highlight the scope and impact of these disorders. The review examines various risk factors associated with postpartum mood disorders, including biological, psychological, and socioeconomic factors. Understanding these risk factors helps identify high-risk populations and guide targeted interventions. Screening and diagnosis of postpartum mood disorders are crucial for early detection and intervention. The article provides an overview of screening tools, highlights the challenges in diagnosis, and emphasizes the importance of early identification for better outcomes. Prevention strategies are explored, including antenatal education, psychosocial support programs, and the role of healthcare professionals in promoting preventive measures. Effective prevention interventions and their outcomes are discussed to guide healthcare providers and policymakers in implementing evidence-based strategies. Treatment approaches for postpartum mood disorders include pharmacological interventions, psychotherapy options, alternative and complementary therapies, and multidisciplinary approaches. The article discusses the effectiveness and considerations of each approach, highlighting the importance of individualized care. Challenges and barriers in diagnosing, preventing, and treating postpartum mood disorders are addressed, including stigma, limited access to healthcare services, and gaps in healthcare provider knowledge and training. Recommendations are provided for healthcare professionals and policymakers to overcome these challenges and improve outcomes. The review concludes by highlighting the need for future research, innovations in prevention and treatment approaches, and collaborative efforts in the field of postpartum mood disorders. Promising areas for research are identified, including long-term outcomes, understanding risk factors, and cultural considerations. The article emphasizes the importance of interdisciplinary collaboration and stakeholder engagement in advancing the field.

## Introduction and background

Postpartum mood disorders are a significant public health concern affecting numerous women worldwide. The postpartum period, typically considered the first year after childbirth, is a time of immense physical, hormonal, and psychological changes for new mothers. While many women experience various emotions, some may develop more severe and persistent mood disorders, such as postpartum depression, anxiety, and psychosis [1,2]. Postpartum mood disorders refer to a spectrum of mental health conditions that can occur following childbirth. These disorders are distinct from the commonly experienced "baby blues," which are mild and transient mood changes. Postpartum depression, the most prevalent form of postpartum mood disorder, is characterized by persistent sadness, worthlessness, and loss of interest or pleasure in daily activities. Postpartum anxiety involves excessive worry, restlessness, and intrusive thoughts, while postpartum psychosis is a rare but severe condition characterized by hallucinations, delusions, and disorganized thinking [2,3].

Postpartum mood disorders have far-reaching implications for both the mother and her family. They can significantly impact the mother's ability to care for herself and her baby, disrupt maternal-infant bonding, and interfere with the overall well-being of the family unit. Moreover, untreated postpartum mood disorders can have long-term effects on the mother's mental health, with potential implications for subsequent pregnancies and parenting [4].

This review article aims to provide a comprehensive overview of postpartum mood disorders, focusing specifically on insights into their diagnosis, prevention, and treatment. By examining current research and literature, this review aims to enhance understanding of the complex nature of postpartum mood disorders, shed light on the various factors contributing to their development, and highlight effective strategies for identifying, preventing, and treating them. This review aims to consolidate knowledge and evidence on postpartum mood disorders, including their classification, risk factors, screening and diagnosis methods, prevention strategies, and treatment approaches. By synthesizing this information, we hope to provide healthcare professionals, researchers, and policymakers with valuable insights to guide clinical practice, inform policy development, and improve the overall support and care for women experiencing postpartum mood disorders.

## Review

Classification and types of postpartum mood disorders

Definition and Diagnostic Criteria

Postpartum mood disorders encompass a range of mental health conditions that occur in the postpartum period. These disorders are characterized by depressive, anxious, or psychotic symptoms that significantly impair a woman's functioning and well-being. Diagnostic criteria for these disorders are often derived from established psychiatric classification systems, such as the Diagnostic and Statistical Manual of Mental Disorders Fifth Edition (DSM-5) or the International Classification of Diseases 11th Revision (ICD-11) [5].

Differentiating Between Postpartum Depression, Postpartum Anxiety, and Postpartum Psychosis

Postpartum depression: Postpartum depression is the most common type of postpartum mood disorder. It involves persistent sadness, hopelessness, and a loss of interest or pleasure in activities. Additional symptoms may include changes in appetite and sleep patterns, fatigue, irritability, difficulty concentrating, and thoughts of self-harm or suicide [6].

Postpartum anxiety: Postpartum anxiety is characterized by excessive worry, restlessness, and intrusive thoughts related to the baby's well-being or the mother's ability to care for the baby. Women with postpartum anxiety may experience panic attacks, racing thoughts, irritability, and physical symptoms, such as shortness of breath or heart palpitations [7].

Postpartum psychosis: Postpartum psychosis is a rare but severe condition characterized by hallucinations, delusions, and disorganized thinking. Women experiencing postpartum psychosis may exhibit rapid mood swings, confusion, agitation, and impulsivity. It requires immediate medical attention due to the potential risk of harm to the mother or the baby [5].

Exploring the Prevalence and Incidence Rates

Postpartum mood disorders, including postpartum depression, anxiety, and psychosis, are significant mental health concerns affecting women worldwide during the postpartum period. The prevalence rates of these disorders vary depending on the specific disorder and the population under study [8].

Postpartum depression: Estimates suggest that postpartum depression affects approximately 10-20% of women after childbirth. However, it is important to note that these rates may vary across different regions and cultures. Certain factors may increase the risk of developing postpartum depression, such as a previous history of depression, low social support, or exposure to stressful life events. Women with these risk factors may have a higher likelihood of experiencing postpartum depression [9].

Postpartum anxiety: Postpartum anxiety is another common mood disorder, impacting approximately 10-15% of women postpartum. It often co-occurs with postpartum depression, and the two conditions may share similar risk factors. Women with postpartum anxiety may experience excessive worry, fear, and restlessness, which can significantly interfere with their daily functioning and well-being [10].

Postpartum psychosis: Postpartum psychosis is a relatively rare but severe condition, occurring in approximately one to two per 1,000 women who have recently given birth. It typically emerges within the first few weeks after delivery and requires immediate medical intervention. Hallucinations, delusions, confusion, and disorganized behavior characterize postpartum psychosis. It poses a significant risk to the affected woman and her infant, necessitating urgent treatment and support [11].

Understanding the prevalence and incidence rates of postpartum mood disorders is crucial for identifying at-risk populations and implementing appropriate screening strategies. By recognizing the prevalence of these disorders, healthcare professionals can better target resources, provide timely interventions, and offer necessary support and treatment to women experiencing postpartum mood disorders. Early identification and intervention can help alleviate symptoms, reduce the impact on maternal well-being, and promote optimal outcomes for both the mother and infant [12].

Risk factors for postpartum mood disorders

Biological Factors (Hormonal Changes and Genetics)

Hormonal changes: The postpartum period is characterized by dramatic hormonal changes, including a rapid decline in estrogen and progesterone levels. These hormonal fluctuations are believed to be significant in developing postpartum mood disorders. Estrogen and progesterone have known effects on neurotransmitter systems in the brain, such as serotonin and dopamine, which regulate mood. The sudden drop in hormone levels after childbirth may disrupt the delicate balance of these neurotransmitters, potentially contributing to the onset of postpartum depression, anxiety, or other mood disorders. However, the exact mechanisms by which hormonal changes influence mood during the postpartum period are not yet fully understood and continue to be a subject of ongoing research [13].

Genetic predisposition: Emerging evidence suggests a genetic predisposition to postpartum mood disorders. Women with a family history of depression or other mood disorders may have an increased susceptibility to experiencing postpartum depression or anxiety. Studies have identified specific genes and genetic variations associated with an increased risk of developing these disorders. Genetic factors interact with environmental and hormonal influences, contributing to the complex interplay of factors contributing to postpartum mood disorders. Understanding these disorders' genetic underpinnings can help identify individuals at higher risk and develop targeted prevention and treatment strategies [14].

Psychological Factors (History of Mental Health Issues, Stress, and Lack of Support)

Previous history of mental health issues: Women with a history of depression, anxiety, or other mental health disorders are at a higher risk of experiencing postpartum mood disorders. In addition, a history of postpartum depression in a previous pregnancy increases the likelihood of recurrence in subsequent pregnancies. A pre-existing mental health condition amplifies the vulnerability to postpartum mood disorders, emphasizing the need for proactive screening and support [15].

Perinatal stressors: The period is marked by significant life changes and stressors that can contribute to developing postpartum mood disorders. High-stress levels during pregnancy or postpartum, such as financial difficulties, relationship problems, or major life events, increase the risk. The combination of hormonal changes, adjustment to the demands of motherhood, and external stressors can overwhelm women and make them more susceptible to mood disorders [15].

Lack of social support: Adequate social support is crucial postpartum, as it can significantly impact maternal mental health. Insufficient emotional support from partners, family members, or friends can contribute to feelings of isolation and exacerbate the risk of postpartum mood disorders. In addition, the absence of practical assistance in caring for the newborn can increase the burden on the mother, adding to her stress and emotional strain [16].

Socioeconomic Factors (Socioeconomic Status and Cultural Influences)

Socioeconomic status: Socioeconomic factors are crucial in developing postpartum mood disorders. Women from lower socioeconomic backgrounds often face additional stressors that can contribute to the risk of these disorders. Financial constraints, limited access to healthcare services, and inadequate social support systems can all contribute to increased vulnerability. The financial burden of caring for newborns and limited resources for adequate prenatal and postnatal care can heighten stress levels and impact mental well-being. The lack of social support networks and resources may lead to feelings of isolation and exacerbate the symptoms of postpartum mood disorders. Furthermore, the challenges associated with balancing work and family responsibilities in low-income households can increase stress levels and the likelihood of experiencing these disorders [17].

Cultural influences: Cultural beliefs and practices surrounding childbirth and motherhood can significantly influence the risk of postpartum mood disorders. Cultural norms related to expressing emotions, seeking help, and seeking treatment for mental health issues can impact how individuals perceive and cope with postpartum mood disorders. In some cultures, stigmatization or taboo may be associated with acknowledging and discussing mental health concerns, including postpartum mood disorders. This stigma can prevent women from seeking help or openly discussing their experiences, leading to underreporting and delayed intervention. Cultural variations in social support networks and family structures can also affect the availability and quality of support during the postpartum period. The level of family involvement, community support, and cultural expectations surrounding maternal roles can influence the level of stress and support experienced by women, thereby impacting the likelihood of developing postpartum mood disorders [18].

Identifying High-Risk Populations

Identifying high-risk populations is crucial for healthcare providers to target interventions and support services for postpartum mood disorders effectively. While these disorders can affect women from all backgrounds, certain groups may be at higher risk due to specific factors. Recognizing these risk factors enables healthcare professionals to effectively implement preventive measures, early detection, and intervention strategies [19].

Women with a history of mental health issues or previous postpartum mood disorders: Women who have experienced mental health issues, such as depression or anxiety, or have a history of postpartum mood disorders are at a higher risk. Healthcare providers should pay special attention to these individuals and provide appropriate support postpartum [20].

Women with a family history of mood disorders: A family history of mood disorders, such as depression or bipolar disorder, increases the susceptibility to postpartum mood disorders. Recognizing this familial risk factor can help healthcare professionals identify women needing closer monitoring and support [21].

Women experiencing significant life stressors or lacking social support: Stressful life events, such as financial difficulties, relationship problems, or lack of social support, can contribute to developing postpartum mood disorders. Healthcare providers should be attentive to the presence of these stressors and provide resources and interventions to mitigate their impact [8].

Women from lower socioeconomic backgrounds with limited resources and access to healthcare: Women from lower socioeconomic backgrounds often face challenges related to limited resources, inadequate healthcare access, and socioeconomic disparities. These factors can increase the risk of postpartum mood disorders. Healthcare providers must address these barriers and ensure equitable access to support and care [22].

Women from minority or marginalized communities may face additional cultural or societal challenges: Women from minority or marginalized communities may encounter cultural or societal challenges contributing to the risk of postpartum mood disorders. Language barriers, cultural stigma, and lack of culturally competent care can impact their access to appropriate support and treatment. Healthcare providers should be aware of these challenges and strive to provide culturally sensitive care to meet the specific needs of these populations [23]. Understanding these risk factors and identifying high-risk populations allows healthcare professionals to tailor their approaches to address postpartum mood disorders effectively. By implementing targeted interventions, early detection measures, and culturally sensitive care, healthcare providers can make significant strides in supporting and improving the mental health outcomes of women at risk for postpartum mood disorders.

Screening and diagnosis of postpartum mood disorders

Overview of Screening Tools and Questionnaires

Screening for postpartum mood disorders is an essential step in the early detection and intervention of these conditions. Numerous screening tools and questionnaires have been developed to aid healthcare providers in identifying women who may be at risk or experiencing symptoms of postpartum mood disorders. These tools facilitate the systematic assessment of symptoms, allowing for timely intervention and support. Here are three commonly used screening tools:

Edinburgh Postnatal Depression Scale (EPDS): The EPDS is a widely utilized self-report questionnaire designed to assess the presence and severity of depressive symptoms in postpartum women. It consists of 10 items that cover a range of emotions and behaviors associated with depression. The EPDS can be administered as early as the first few weeks after childbirth and has been extensively validated for its effectiveness in detecting postpartum depression. A cut-off value of 11 or higher on the EPDS demonstrated the optimal balance between sensitivity and specificity, maximizing their combined effectiveness. Meanwhile, a cut-off value of 13 or higher showed reduced sensitivity but increased specificity. When identifying pregnant and postpartum women with elevated symptom levels, a cut-off of 13 or higher would be appropriate. However, lower cut-off values could be employed if the goal is to minimize the occurrence of false negatives and capture a larger portion of patients who meet the diagnostic criteria [24].

Postpartum Depression Screening Scale (PDSS): The PDSS is a comprehensive self-report measure specifically developed to assess symptoms of postpartum depression. This scale encompasses a broader spectrum of emotional and physical symptoms commonly experienced postpartum. It comprehensively evaluates the severity and nature of depressive symptoms, enabling healthcare providers to identify women who may require further assessment or intervention [25].

Generalized Anxiety Disorder 7 (GAD-7): Although not specifically designed for postpartum mood disorders, the GAD-7 is frequently used as a screening tool for anxiety symptoms, which often co-occur with postpartum depression. The GAD-7 consists of seven items that assess the severity of generalized anxiety symptoms. It provides valuable information to healthcare providers regarding the presence and severity of anxiety symptoms, aiding in identifying women who may require additional assessment or support [26]. These screening tools, among others, serve as valuable resources for healthcare providers in identifying and monitoring postpartum mood disorders, and it have a sensitivity of 89% and a specificity of 82%. By implementing routine screening using validated tools, healthcare professionals can promptly identify women at risk or experiencing symptoms, allowing for early intervention and appropriate treatment. It is important to note that a comprehensive clinical evaluation should accompany screening to ensure accurate diagnosis and tailor the treatment approach to each woman's needs.

Challenges and Limitations in Diagnosing Postpartum Mood Disorders

Overlapping symptoms: One of the challenges in diagnosing postpartum mood disorders is the overlap of symptoms with normal postpartum adjustment. The emotional and physical changes experienced after childbirth can be similar to the symptoms of postpartum mood disorders, such as sadness, fatigue, and changes in appetite. This similarity can make it difficult for healthcare providers to differentiate between normal adjustment and a mood disorder, potentially leading to underdiagnosis or misdiagnosis [27].

Variability of presentation: Postpartum mood disorders can manifest in various ways, and the presentation can vary greatly among individuals. Symptoms may differ in terms of severity, duration, and specific manifestation. For example, some women may experience primarily depressive symptoms, while others may have predominantly anxious symptoms or even symptoms of psychosis. This variability adds complexity to the diagnostic process, as healthcare providers must carefully evaluate the individual's unique symptomatology to make an accurate diagnosis [28].

Lack of awareness and stigma: There is a general lack of awareness and understanding surrounding postpartum mood disorders among the general population and healthcare providers. This lack of awareness can lead to the under-recognition of symptoms and delayed help-seeking. In addition, the stigma associated with mental health issues can create barriers for women to seek help or openly discuss their symptoms. The fear of being judged as inadequate mothers or facing societal stigma may prevent women from reporting their symptoms, further hindering the diagnostic process. Healthcare providers are crucial in creating a supportive and non-judgmental environment that encourages open communication and empowers women to seek help [29].

Addressing these challenges requires improved education and awareness about postpartum mood disorders among healthcare providers and the general public. Enhanced training for healthcare professionals can help them recognize the nuances of symptom presentation and differentiate between normal adjustment and mood disorders. In addition, efforts should be made to reduce the stigma associated with mental health, creating a safe space for women to discuss their experiences without fear of judgment. By addressing these challenges, healthcare providers can improve the accuracy of diagnoses and ensure that women receive appropriate support and treatment for postpartum mood disorders [30].

Importance of Early Detection and Intervention

Early detection and intervention are crucial in effectively managing postpartum mood disorders. The timely identification of symptoms allows for prompt intervention, significantly reducing the potential negative impact on the mother, baby, and family. Moreover, early intervention can potentially prevent the escalation of symptoms and the development of more severe or chronic conditions [2].

One key benefit of early detection is the opportunity for enhanced support and education. By identifying women who are at risk or experiencing symptoms of postpartum mood disorders, healthcare providers can offer appropriate support, education, and resources. This empowers women and their families to understand the condition better and develop effective coping strategies. By providing timely information and support, healthcare professionals can contribute to the overall well-being of the affected individuals and their families [29].

Another advantage of early detection is the ability to develop tailored treatment plans. Healthcare providers, armed with the knowledge of early symptoms, can create individualized treatment strategies that address the specific needs of each woman. These treatment plans may involve a combination of psychological interventions, pharmacotherapy, and social support, among other approaches. By initiating treatment at an early stage, healthcare professionals can improve the likelihood of positive outcomes and facilitate the recovery process [31].

Prevention strategies for postpartum mood disorders

Antenatal Education and Preparation

Prenatal education on postpartum mood disorders: Expectant mothers and their families can greatly benefit from comprehensive education about postpartum mood disorders. This education aims to increase awareness; provide information about the signs, symptoms, and risk factors associated with these disorders; and emphasize the importance of early recognition and intervention. Prenatal classes, led by healthcare professionals or trained educators, offer a structured platform to deliver this education. These classes may cover baby blues, postpartum depression, anxiety, and psychosis. Informational materials, such as pamphlets, brochures, or online resources, can also disseminate important information to expectant mothers and their families. By educating on postpartum mood disorders during the prenatal period, women can be better prepared to identify potential challenges and seek help if needed [32].

Birth and postpartum planning: Assisting women in developing birth and postpartum plans can help alleviate stress and promote a sense of control and preparedness. Birth planning involves discussions between expectant mothers and their healthcare providers about preferences for labor and delivery, pain management options, and potential complications. By actively involving women in decision-making processes, birth planning empowers them and reduces uncertainty and anxiety. Postpartum planning focuses on the period following childbirth and encompasses various aspects, including emotional well-being. During postpartum planning, expectant mothers are encouraged to consider their support networks, including family, friends, and healthcare professionals. Identifying individuals who can provide emotional and practical support during the postpartum period. In addition, exploring coping strategies, self-care practices, and resources for mental health support can be incorporated into the postpartum plan. By engaging in birth and postpartum planning, women can feel more prepared and supported during the transitional period after giving birth [33].

Psychosocial Support Programs

Psychosocial support programs are crucial in preventing and managing postpartum mood disorders. These programs encompass a range of interventions designed to provide emotional support, education, and coping strategies for women during the postpartum period. Two key components of psychosocial support programs are peer support groups and counseling/therapy services [34].

Peer support groups: Peer support programs are designed to create a safe and non-judgmental space where women can connect with others who have experienced or are currently experiencing postpartum mood disorders. These groups offer valuable emotional support, validation, and information sharing. By interacting with peers who empathize with their experiences, women can feel understood, reduce feelings of isolation, and gain insights into coping strategies and self-care practices. Peer support groups may be facilitated by trained facilitators or led by peers who have successfully navigated their postpartum mood disorders. These groups allow women to express their feelings, share their challenges, and learn from each other's experiences, fostering community and empowerment [35].

Counseling and therapy: Counseling and therapy services, such as cognitive-behavioral therapy (CBT) or interpersonal therapy (IPT), are essential components of psychosocial support programs. These interventions aim to provide women with professional guidance and support in managing postpartum mood disorders. CBT identifies and modifies negative thought patterns and behaviors, helping women develop healthier coping mechanisms and adaptive strategies. Meanwhile, IPT emphasizes improving interpersonal relationships and resolving conflicts, recognizing the impact of social support on mental well-being. Counseling and therapy sessions offer a structured and personalized approach to addressing the unique challenges faced by women with postpartum mood disorders. They provide a supportive environment where women can explore their emotions, learn effective communication skills, and develop strategies to manage stress and anxiety. These interventions empower women to take an active role in their mental health and equip them with the tools to navigate the postpartum period more effectively [36].

Psychosocial support programs, including peer support groups and counseling/therapy services, are valuable components of comprehensive care for women with postpartum mood disorders. By offering emotional support, promoting self-care practices, and providing evidence-based interventions, these programs contribute to the prevention and management of postpartum mood disorders, ultimately improving women's overall well-being during this vulnerable period [37].

Role of Healthcare Professionals in Prevention Efforts

Healthcare professionals play a crucial role in the prevention of postpartum mood disorders. Their involvement encompasses various aspects, including screening, early intervention, and postpartum followup care. By actively engaging in these areas, healthcare professionals can contribute significantly to the well-being of women during the postpartum period [9].

Screening and early intervention: Through regular screenings, healthcare professionals are well-positioned to identify women at risk or experiencing early signs of postpartum mood disorders. By implementing validated screening tools and assessment measures, healthcare providers can effectively identify women at higher risk or experiencing symptoms of postpartum mood disorders. Early intervention is key to preventing the escalation of symptoms and improving outcomes. Healthcare professionals can provide essential information about postpartum mood disorders, offer support and validation, and refer women to appropriate mental health specialists or support services. By offering timely intervention, healthcare professionals can help women access the necessary resources and support to manage their mental health effectively [9].

Postpartum followup care: Maintaining regular postpartum care, including routine check-ups and assessments, is crucial in supporting women's mental health. Healthcare professionals can be vital in monitoring women's mental well-being during this critical postpartum period. Healthcare providers can identify early signs of postpartum mood disorders by conducting comprehensive assessments and evaluating for emerging symptoms or challenges. Regular followup care allows for ongoing support, counseling, and guidance, ensuring that women receive appropriate care and interventions tailored to their needs. This continuity of care enables healthcare professionals to track progress, make necessary adjustments to treatment plans, and provide additional resources or referrals as required [38].

The role of healthcare professionals in prevention efforts extends beyond mere detection and assessment. They also play a crucial role in educating women and their families about postpartum mood disorders, dispelling myths, and reducing stigma. By promoting awareness, healthcare professionals can encourage open discussions, fostering an environment where women feel comfortable seeking help and support [39].

Identifying Effective Prevention Interventions and Their Outcomes

Identifying effective prevention interventions and evaluating their outcomes is critical to addressing postpartum mood disorders. Several interventions have shown promise in preventing these disorders and improving maternal mental health outcomes [40].

Effectiveness of psychoeducation: Numerous studies have demonstrated the effectiveness of psychoeducation programs delivered during pregnancy or early postpartum. These programs aim to provide expectant and new mothers with information, support, and skills training to enhance their emotional well-being and equip them with coping strategies. Psychoeducation programs have been shown to reduce the incidence and severity of postpartum mood disorders, enhance maternal self-efficacy, and promote early identification and help-seeking behaviors [41].

Home-visiting programs: Home-visiting interventions conducted by trained professionals, such as nurses or social workers, have shown promise in preventing postpartum mood disorders. These programs involve regular home visits during pregnancy and the early postpartum period to provide support, education, and assistance with various maternal and infant well-being aspects. Home-visiting programs focus on enhancing parenting skills, promoting breastfeeding, and addressing maternal mental health. Studies have indicated that such interventions can improve maternal mental health outcomes, increase social support, and reduce stress and depressive symptoms [42].

Collaborative care models: Collaborative care models involve a multidisciplinary approach to addressing postpartum mood disorders. These models typically include healthcare providers, mental health specialists, and social support services working collaboratively to meet the needs of women with or at risk of these disorders. Collaborative care models emphasize comprehensive assessment, individualized treatment planning, and regular followup. Research has shown positive outcomes with these models, including reduced symptom severity, increased treatment adherence, improved quality of life, and enhanced maternal mental health [43].

Treatment approaches for postpartum mood disorders

Pharmacological Interventions (Antidepressants and Anxiolytics)

Antidepressants: Selective serotonin reuptake inhibitors (SSRIs) are frequently recommended as the initial pharmacological treatment for postpartum depression. These medications work by selectively inhibiting the reuptake of serotonin, a neurotransmitter involved in mood regulation. By increasing serotonin levels in the brain, SSRIs can help alleviate depressive symptoms. It is important to note that using antidepressants during breastfeeding requires careful consideration. While some SSRIs have been deemed relatively safe, potential risks and benefits should be evaluated in consultation with a healthcare professional to make an informed decision that ensures the well-being of both the mother and infant [44].

Anxiolytics: In cases where anxiety symptoms are prominent in postpartum mood disorders, anxiolytic medications may be prescribed to manage anxiety disorders. These medications reduce excessive worry, restlessness, and anxiety-related panic symptoms. Anxiolytics can help alleviate anxiety's distressing and disruptive effects, allowing individuals to cope better. Similar to antidepressants, the use of anxiolytics during breastfeeding requires careful consideration and consultation with a healthcare professional to assess potential risks and benefits. The selection of anxiolytics should be based on the individual's needs and overall health situation [45].

Psychotherapy Options (CBT and IPT)

CBT: CBT is a widely recognized and evidence-based psychotherapy approach to treating postpartum mood disorders. CBT focuses on identifying and challenging negative thought patterns and behaviors contributing to emotional distress. Through this therapy, individuals learn to recognize and modify unhelpful thoughts, beliefs, and attitudes, which can alleviate symptoms of postpartum depression, anxiety, and other mood disorders. CBT equips individuals with coping strategies, problem-solving skills, and relaxation techniques to manage stress, improve mood, and enhance overall emotional well-being. The collaborative nature of CBT allows individuals to actively participate in their treatment actively, fostering a sense of empowerment and control over their emotions [46].

IPT: IPT is another well-established psychotherapy approach for treating postpartum mood disorders. IPT addresses interpersonal problems and conflicts that may contribute to developing or exacerbating symptoms. It recognizes the impact of social relationships on an individual's mental health and aims to improve communication, interpersonal relationships, and social support networks. IPT typically involves exploring the individual's relationships and identifying difficulties, such as role transitions, unresolved grief, interpersonal disputes, or social isolation. By addressing these interpersonal issues, IPT helps individuals develop healthier ways of relating to others, enhancing their overall well-being and reducing symptoms of postpartum mood disorders [47].

Alternative and Complementary Therapies

Mindfulness-based interventions: Mindfulness-based interventions, such as mindfulness-based cognitive therapy (MBCT) or mindfulness-based stress reduction (MBSR), have gained recognition as effective approaches for addressing postpartum mood disorders. These interventions involve cultivating present-moment awareness, non-judgmental acceptance, and self-compassion. Studies have shown that practicing mindfulness can reduce stress, promote emotional well-being, and enhance overall mental health postpartum. Mindfulness-based interventions have also demonstrated efficacy in preventing relapse in individuals who have experienced previous episodes of postpartum depression or anxiety. By cultivating mindfulness skills, individuals can develop greater resilience and adaptive coping strategies to navigate the challenges associated with postpartum mood disorders [48].

Yoga and exercise: Regular physical exercise, including yoga, is another promising approach for managing postpartum mood disorders. Physical exercise has numerous mental health benefits, including improved mood, reduced anxiety, and enhanced overall well-being. Yoga, in particular, combines physical movement, breath control, and mindfulness, offering a holistic approach to improving mental and physical health. Yoga practice during the postpartum period has positively affected depressive symptoms, anxiety reduction, and sleep quality. The gentle movements and emphasis on relaxation and body awareness in yoga can help alleviate stress and promote a sense of calm and balance. Moreover, participating in group exercise classes or engaging in physical activities with other postpartum individuals can provide social support and a sense of community, which is beneficial for psychological well-being [49].

Multidisciplinary Approaches for Comprehensive Care

Collaborative care models: Collaborative care models are essential in managing postpartum mood disorders. These models emphasize the coordination and integration of healthcare providers, mental health specialists, and support services to deliver comprehensive care to women in need. By working together, these professionals ensure that all aspects of treatment are addressed, including medication management, therapy, and social support. The collaborative care approach recognizes that postpartum mood disorders require a multidimensional approach, and by pooling their expertise, the care team can develop individualized treatment plans tailored to each woman's specific needs. This model also facilitates communication and collaboration among different healthcare providers, enhancing the continuity and effectiveness of care. By adopting a collaborative care model, women with postpartum mood disorders can receive holistic and coordinated support, improving outcomes and long-term recovery [50].

Postpartum support programs: Postpartum support programs are vital in providing comprehensive assistance to women experiencing postpartum mood disorders. These programs offer a range of services that aim to address the complex needs of women during this challenging period. Counseling services provide a safe and supportive environment where women can express their emotions, discuss their concerns, and receive professional guidance. Support groups bring together individuals who share similar experiences, fostering a sense of community and validation. Educational resources offer valuable information about postpartum mood disorders, coping strategies, and self-care techniques. Case management ensures that women receive personalized care coordination, helping them navigate the healthcare system and access appropriate resources. Postpartum support programs recognize the importance of addressing the emotional, psychological, and practical aspects of postpartum mood disorders. These programs provide comprehensive services to empower women to seek help, reduce isolation, and promote their recovery and overall well-being [9].

Challenges and barriers to diagnosis, prevention, and treatment

Stigma and Cultural Factors

The stigma surrounding mental health: Stigma refers to the negative attitudes, beliefs, and stereotypes associated with mental health conditions, including postpartum mood disorders. Women experiencing these disorders may face societal judgment, fear of being labeled as inadequate or unfit mothers, or concerns about the perceived social stigma surrounding mental health. Consequently, they may hesitate to seek help or openly discuss their symptoms due to the fear of being stigmatized or facing discrimination. The presence of stigma can contribute to a significant barrier to accessing timely diagnosis and treatment for postpartum mood disorders. By addressing stigma through education, awareness campaigns, and promoting open dialogue, we can encourage women to seek help without fear of judgment, thus facilitating early intervention and support [51].

Cultural factors: Cultural beliefs, values, and practices influence how postpartum mood disorders are perceived, recognized, and addressed within different cultural contexts. Cultural norms surrounding motherhood, gender roles, and emotions are crucial in shaping individuals' understanding and acceptance of these disorders. In some cultures, there may be limited awareness or acknowledgment of postpartum mood disorders, leading to underreporting and inadequate support for affected women. Additionally, cultural expectations and pressure to fulfill specific roles and responsibilities may create additional stressors for new mothers, exacerbating the risk of developing postpartum mood disorders. Recognizing and addressing cultural factors is essential for providing culturally sensitive care, tailoring interventions to specific cultural contexts, and promoting inclusivity in diagnosing, preventing, and treating postpartum mood disorders. By engaging with diverse communities and considering cultural perspectives, healthcare professionals can ensure that their approaches align with the needs and values of the individuals they serve, ultimately facilitating better outcomes and reducing disparities in care [18].

Access to Healthcare Services

Availability and affordability: Limited access to healthcare services, particularly in underserved areas, can pose a significant barrier to the diagnosis, prevention, and treatment of postpartum mood disorders. In many regions, healthcare facilities specializing in postpartum mental health may be scarce or inaccessible, leaving women without the necessary resources and support. Financial constraints and a lack of insurance coverage can further hinder access to essential care. The high costs associated with diagnostic assessments, therapy sessions, and medications may deter individuals from seeking help or impede their ability to receive timely and adequate treatment. Addressing these barriers requires strategic interventions, such as improving the availability of mental health services in underserved areas, reducing financial burdens through affordable healthcare options, and advocating for comprehensive insurance coverage for postpartum mental health [52].

Postpartum care gaps: Discontinuity in postpartum care and inadequate followup appointments can contribute to missed opportunities for early detection and intervention in postpartum mood disorders. The postpartum period is critical for monitoring and addressing women's mental health, yet the healthcare system often fails to provide adequate support during this phase. Limited postpartum visits or a lack of routine mental health screenings may result in undiagnosed or untreated mood disorders. Improving access to postpartum care is vital, including regular followup appointments and incorporating mental health assessments as a routine part of postpartum check-ups. Bridging the gaps in postpartum care can ensure that women receive appropriate support, early intervention, and timely treatment for postpartum mood disorders, improving outcomes for both mothers and their families. Efforts should be made to educate healthcare providers about the importance of postpartum mental health and the implementation of evidence-based guidelines to facilitate comprehensive postpartum care [2].

Gaps in Healthcare Provider Knowledge and Training

Lack of screening and diagnostic tools: One of the challenges in addressing postpartum mood disorders is the lack of standardized screening and diagnostic tools. Some healthcare providers may not be familiar with or have access to validated screening tools specifically designed for postpartum mood disorders. This can result in underdiagnosis or misdiagnosis, as symptoms may be overlooked or attributed to other factors. In addition, healthcare providers may lack training or knowledge in recognizing these disorders' subtle signs and symptoms, further hindering accurate diagnosis. Improved education and awareness among healthcare professionals about the availability and importance of validated screening tools can help ensure early detection and appropriate diagnosis of postpartum mood disorders [53].

Limited awareness of treatment options: Another challenge lies in the limited awareness among healthcare providers regarding the full range of evidence-based treatment options for postpartum mood disorders. While pharmacological interventions, such as antidepressants and anxiolytics, are commonly used, there may be a lack of awareness about other effective treatment modalities, such as psychotherapy options (e.g., CBT and IPT) and alternative and complementary therapies (e.g., yoga and mindfulness). This limited awareness may lead to suboptimal or inadequate treatment for affected individuals, as they may not receive the most appropriate interventions for their needs. Enhancing education and training for healthcare professionals regarding the various treatment approaches available can help ensure that individuals with postpartum mood disorders receive comprehensive and individualized care, improving their chances of recovery and well-being [54].

Addressing These Challenges for Better Outcomes

Education and awareness campaigns: Promoting public education and awareness about postpartum mood disorders is crucial in reducing stigma and increasing early recognition and help-seeking behaviors. Targeted campaigns can be developed to educate healthcare providers, the general public, and specific cultural communities about the signs, symptoms, and available resources for postpartum mood disorders. By increasing knowledge and understanding, these campaigns can improve identification and support for affected individuals [55].

Integration of mental health services: Integrating mental health services into routine antenatal and postpartum care is essential for early detection and intervention. Healthcare providers should receive training on recognizing the symptoms of postpartum mood disorders, conducting appropriate assessments, and making timely referrals to mental health professionals. Collaborative care models, where obstetricians, midwives, and mental health providers work together, can ensure comprehensive support and treatment for women experiencing postpartum mood disorders [56].

Improving access to care: Access to affordable and comprehensive healthcare services, including mental health support, should be prioritized to address postpartum mood disorders. Efforts should be made to expand healthcare coverage and ensure that specialized services for postpartum mental health are readily available. In addition, telehealth options can help overcome barriers, such as geographical distance, allowing women in underserved areas to conveniently access the care they need [57].

Professional training and support: Healthcare professionals are critical in identifying, managing, and treating postpartum mood disorders. Providing them with ongoing training and support can enhance their competence and confidence in addressing these conditions effectively. Training programs should focus on evidence-based assessment tools, treatment approaches, and interventions for postpartum mood disorders. In addition, fostering a supportive environment and offering supervision and consultation services can help healthcare professionals navigate complex cases and enhance the quality of care provided to women with postpartum mood disorders [58].

Future directions and research implications

Promising Areas for Future Research

Long-term outcomes: Further research is needed to explore postpartum mood disorders' long-term outcomes and consequences on women's mental health, child development, and family dynamics. Longitudinal studies can provide valuable insights into the trajectory of these disorders and their impact over time.

Understanding risk factors: More research is needed to elucidate the complex interplay of biological, psychological, and socioenvironmental factors that contribute to developing postpartum mood disorders. Identifying specific risk factors can inform targeted prevention strategies and interventions.

Cultural considerations: Future research should aim to understand better the influence of cultural factors on the recognition, experience, and treatment of postpartum mood disorders. Culturally sensitive approaches can help address barriers and enhance outcomes for diverse populations.

Innovations in Prevention and Treatment Approaches

Technology-based interventions: Exploring the use of digital health technologies, such as mobile applications and online platforms, for the prevention, screening, and treatment of postpartum mood disorders shows promise. These interventions can increase accessibility, provide psychoeducation, and offer support in a convenient and user-friendly manner.

Personalized interventions: Advancements in precision medicine and the development of biomarkers may lead to personalized prevention and treatment approaches for postpartum mood disorders. Tailoring interventions based on individual characteristics and needs can optimize outcomes and reduce the burden of these disorders.

Integrated care models: Implementing integrated care models that bring together obstetric care, mental health services, and social support can improve coordination and ensure holistic care for women experiencing postpartum mood disorders. These models can enhance collaboration among healthcare providers and streamline access to comprehensive services.

Importance of Collaborative Efforts and Interdisciplinary Research

Interdisciplinary research: Collaboration between researchers from various disciplines, including psychology, psychiatry, obstetrics, social work, and public health, is crucial for advancing our understanding of postpartum mood disorders. Interdisciplinary approaches can lead to comprehensive insights and innovative solutions.

Stakeholder engagement: Involving key stakeholders, such as women with lived experiences, healthcare providers, policymakers, and community organizations, in research and practice efforts is essential. Their perspectives and input can guide the development of relevant and effective interventions and policies.

Knowledge dissemination: Dissemination of research findings and evidence-based guidelines to healthcare professionals, policymakers, and the general public is vital to bridge the gap between research and practice. Education and awareness initiatives can promote informed decision-making, reduce stigma, and improve the quality of care for postpartum mood disorders.

## Conclusions

This review article provides valuable insights into diagnosing, preventing, and treating postpartum mood disorders. We have explored the background information, significance, and purpose of understanding these disorders. The classification and types of postpartum mood disorders, prevalence, and incidence rates were discussed, highlighting the need for accurate identification and differentiation among different disorders. We delved into the risk factors associated with postpartum mood disorders, including biological, psychological, and socioeconomic factors. Recognizing these risk factors can aid in targeted interventions and support for high-risk populations. In addition, we examined the screening and diagnosis process, emphasizing the importance of early detection and intervention to mitigate the negative impact on maternal well-being. The review also explored prevention strategies, such as antenatal education, psychosocial support programs, and the role of healthcare professionals in promoting preventive measures. By addressing risk factors, promoting mental health literacy, and fostering supportive environments, we can strive to prevent or minimize the occurrence and severity of postpartum mood disorders. Treatment approaches were thoroughly discussed, including pharmacological interventions, psychotherapy options, alternative and complementary therapies, and multidisciplinary approaches for comprehensive care. Highlighting the effectiveness and considerations of each approach can guide healthcare professionals in developing individualized treatment plans and promoting holistic recovery.
